# Evolution of sexual mimicry in the orchid subtribe orchidinae: the role of preadaptations in the attraction of male bees as pollinators

**DOI:** 10.1186/1471-2148-8-27

**Published:** 2008-01-28

**Authors:** Florian P Schiestl, Salvatore Cozzolino

**Affiliations:** 1 Institute of Systematic Botany, University of Zürich, Zollikerstrasse 107, CH-8008 Zürich, Switzerland; 2 Università degli Studi di Napoli "Federico II", Dipartimento delle Scienze Biologiche, Via Foria 223, 80139 Napoli, Italy

## Abstract

**Background:**

Within the astonishing diversity of orchid pollination systems, sexual deception is one of the most stunning. An example is the genus *Ophrys*, where plants attract male bees as pollinators by mimicking female mating signals. Unsaturated hydrocarbons (alkenes) are often the key signal for this chemical mimicry. Here we investigate the evolution of these key compounds within Orchidinae by mapping their production in flowers of selected species onto their estimated phylogeny.

**Results:**

We found that alkenes, at least in trace amounts, were present in 18 of 20 investigated species together representing 10 genera. Thus, the reconstruction of ancestral state for alkene-production showed that this is a primitive character state in *Ophrys*, and can be interpreted as a preadaptation for the evolution of sexual deception. Four of the investigated species, namely *Ophrys sphegodes*, *Serapias lingua, S. cordigera*, and *Anacamptis papilionacea*, that are pollinated primarily by male bees, produced significantly larger amounts and a greater number of different alkenes than the species pollinated either primarily by female bees or other insects.

**Conclusion:**

We suggest that high amounts of alkenes evolved for the attraction of primarily male bees as pollinators by sensory exploitation, and discuss possible driving forces for the evolution of pollination by male bees.

## Background

Animal-pollinated plants produce floral signals to advertise their rewards, or mimic attractive signals to cheat pollinating animals [1-6]. Floral mimicry can involve imitations of rewarding flowers, egg deposition substrates such as fungi, faeces or rotting meat, or sexual signals. Floral mimicry systems, as well as pollination systems in general, are surprisingly varied, even within single plant families. Orchids are a classic example, famous for their unparalleled diversity of pollination systems [7]. For example, 19 different specialized pollination systems were recognised within 27 investigated species in the genus *Disa *[8]. Orchids have also evolved some of the best known and elaborated pollination systems, such as pollination by fragrance collecting bees [9] or sexual deception [10]. Not surprisingly, evolutionary patterns and mechanisms of such diversity have long puzzled researchers [11].

Plant pollinator interactions often depend on a combination of different signals [12-14]. Whereas floral colour is frequently included in studies on evolutionary patterns of pollination systems, floral scent has been largely ignored. However, floral scent can be the key signal for the attraction of certain pollinator guilds (e.g. nocturnal moths, fragrance-collecting euglossine bees [9,15,16]) and plays a decisive role in the evolution of several mimicry systems (e.g. carrion mimicry, sexual deception) [17-19]. Therefore, a better understanding of the evolution of floral scent is needed to achieve a more conclusive view on the mechanisms and patterns of evolution of pollination systems.

Sexual mimicry, or sexual deception, is well known in European, Australian and Neotropical orchids, and is likely more widely distributed than currently known (summarized in [4]). This pollination system functions primarily through the species-specific imitation of female sex pheromones, in combination with less specific visual and tactile stimuli produced by the flowers. The mechanism of this chemical mimicry was first elucidated by Schiestl et al. [17], who showed that specific patterns of n-alkenes (unsaturated hydrocarbons) play a key role in orchids of the genus *Ophrys *(Orchideae, Orchidinae) for the attraction of male pollinators to the flowers. Alkanes, saturated analogues of n-alkenes, were shown to have a synergistic effect by increasing the intensity of male responses [20].

Both alkanes and alkenes are well-known compounds that have been chemically characterized in several organisms. They are present in plant and insect cuticular wax layers, with the primary function of reducing water evaporation [21-23]. Whereas long chain alkanes are widespread and abundant in plant and insect waxes, alkenes have, up to now, only been found in few species, e.g. in the *Rosa *and *Aloe *spp. perianth, as well as sugar cane wax, and spike wax of *Agropyron intermedium *[24,25]. They generally decrease the melting temperature of the wax layer [26] and increase its permeability [21]. Being present at the outer layer of the plant, alkanes and alkenes are also important in mediating plant herbivore interactions, both through physical properties and semiochemical (i.e. volatile signalling) functions [27,28]. In insects, alkanes and alkenes have similar physiological functions in the wax layer of the cuticle, but also act as pheromones in intraspecific communication, by mediating kin recognition or attraction of the opposite sex [29]. Alkenes are well known as sex pheromone compounds in flies, for example, 9-tricosene is a sex pheromone component of *Musca domestica*, whereas *Drosophila melanogaster *uses 7-tricosene and 7-pentacosene [30]. Among solitary bees, alkanes and alkenes are also the male-attracting pheromones in many species [31]. Thus, there is little doubt that many insect species use alkenes as communication signals, and consequently, there are ample opportunities for plants to mimic such signals to abuse insects as pollinators, such as by "sexual swindle" in *Ophrys *[17].

A powerful approach to trace evolutionary trajectories of pollination systems is their mapping onto phylogenies [32], which has often highlighted their high evolutionary flexibility within evolutionary lineages [8,33]. Fewer studies have combined phylogenies with ecological or physiological studies to investigate the evolution of specific floral traits in relation to their functions, or change of functions [34-39]. Such studies are important for detecting evolutionary changes in the functions of key traits, and can help explain flexibility in evolutionary lineages, as well as adaptive radiations triggered by key innovations.

Although it is clear that *Ophrys *produces alkenes for sexual deception, it is an open question whether alkene production was a key innovation for the evolution of this pollination system, or represents a preadaptation, that primarily had a non-reproductive function in the Orchidinae. This study aims to test these hypotheses by mapping the production, diversity, and amount of alkenes on a phylogeny of the investigated Orchidinae, asking the following question: (1) is the production of alkenes as pheromone-mimics in *Ophrys *a plesiomorphic state for this genus? (2) Is there is any link between alkene-production and pollination syndrome among the investigated species?

## Results

### Occurrence of alkenes

**Figure 1:**
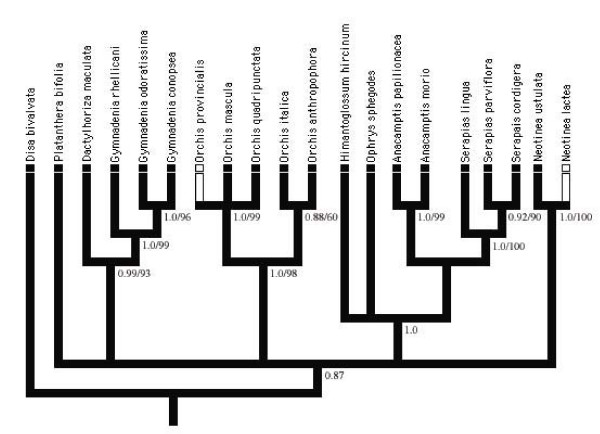
Occurrence of alkenes mapped onto the estimated phylogeny of the studied orchid species. The strict consensus tree was obtained among 6 equally most-parsimonious topologies based on molecular data. Black filled = alkenes present, empty = alkenes absent. Numbers below each branch are the Bayesian a posteriori probabilities/most parsimonious bootstrap of each clade above 50%.

Of 19 European orchid species representing 9 genera (Table 1), n-alkanes (saturated hydrocarbons) of chain lengths 21 – 29 were present in labella of all species, but in varying relative and absolute abundance (Table 2). Odd chain-lengths alkanes with 23 carbon atoms or more were especially abundant (Table 2). Alkenes (unsaturated hydrocarbons), although generally less common, were also widespread among the investigated species (Fig. 1). In total, 17 species produced at least trace amounts of alkenes in their cuticular waxes (Table 2). In only two species (*Orchis provincialis, Neotinea lactea*), no alkenes could be detected. In 11 species, alkenes constituted more than 10% of all analysed compounds (Table 2). Some species (*Serapias lingua*, *S. cordigera*, *Gymnadenia conopsea*, *Neotinea ustulata*) produced 50% or a higher proportion of alkenes in their wax layer. The highest relative abundance of a single alkene was found in *N. ustulata*, where 11-tricosene (C23en) represented 42% of all the compounds found in this species. The South African *Disa bivalvata*, used here as outgroup, produced 81.9 μg (80.6%) alkanes and 19.7 μg (19.4%) alkenes in a single labellum.

**Table 1:** Sampling locations, pollination syndromes, and pollinators of the studied orchid species

Species	Labellum area (mm^2^)*	Sampling location	Pollination syndrome	Predominant pollinator**
*Orchis italica*	64.00	Roccamonfina, I	Food deception	Female bees
*Orchis provincialis*	78.75	Cilento, I	Food deception	Female bees
*Orchis mascula*	78.00	Cilento, I	Food deception	Female bees
*Orchis quadripunctata*	25.50	Cilento, I	Food deception	Long tongued flies
*Orchis (= Aceras) anthropophora*	39.06	Cilento, I	Food reward	Beetles
*Serapias lingua*	126.50	Cilento, I	Sleeping holes	*Ceratina *males
*Serapias cordigera*	396.00	Cilento, I	Sleeping holes	*Eucera *males
*Serapias parviflora*	47.25	Cilento, I	Autogamy	none
*Ophrys sphegodes*	91.00	Roccamonfina, I	Sexual deception	*Andrena *males
*Anacamptis (= Orchis) papilionacea*	148.75	Roccamonfina, I	Food deception?	*Eucera *males
*Anacamptis (= Orchis) morio*	60.00	Vesuvio, I	Food deception	Female bees
*Gymnadenia (= Nigritella) rhellicani*	12.00	Ofenpass, CH	Food reward	Moths
*Gymnadenia conopsea*	11.88	Münstertal, CH	Food reward	Butterflies, moths
*Gymnadenia odoratissima*	10.13	Münstertal, CH	Food reward	Moths
*Dactylorhiza maculata*	50.63	Münstertal, CH	Food deception	Beetles, flies
*Himantoglossum hircinum*	166.25	Glattfelden, CH	Unknown	Female bees
*Platanthera bifolia*	21.94	Glattfelden, CH	Food reward	Moths
*Neotinea (= Orchis) ustulata*	16.82	Wallis, CH	Food deception?	Tachinid flies
*Neotinea lactea*	40.38	Cilento, I	Unknown	Unknown
*Disa bivalvata*	*n.d*.	Cape, ZAR	Sexual deception	Pompilid males

* Calculated from [79], see methods section. ** From [75] and references therein, and [76, 77, 80]

**Table 2:** Relative and total amounts of n-alkanes (straight chain saturated hydrocarbons) and n-alkenes (straight chain unsaturated hydrocarbons) in the investigated orchid species. Compounds are ordered in retention times. Total amounts are given in ng per single labellum

	*Orchis italica*	*Orchis provincialis*	*Orchis mascula*	*Orchis quadripunct*.	*Orchis anthropoph*.	*Serapias lingua*	*Serapias cordigera*	*Serapias parviflora*	*Ophrys sphegodes*	*Anacamptis papilionacea*
**Alkane**										
Heneicosane (C21)	1.71	0.70	0.66	1.19	1.58	1.20	14.58	0.72	5.27	2.04
Docosane (C22)	0.79	0.00	0.00	0.36	0.75	0.29	1.19	0.00	1.76	0.20
Tricosane (C23)	37.05	0.85	5.53	14.86	10.82	6.13	8.01	1.47	28.41	8.35
Tetracosane (C24)	1.98	0.47	1.72	2.21	0.92	1.63	0.58	1.38	2.51	0.49
Pentacosane (C25)	16.88	12.07	39.38	29.62	24.47	14.28	2.72	16.84	11.08	15.79
Hexacosane (C26)	1.64	5.41	2.73	2.63	1.25	2.20	0.26	3.54	0.72	0.63
Heptacosane (C27)	20.65	46.00	35.32	32.51	26.92	15.00	2.62	33.73	5.19	22.07
Octacosane (C28)	1.38	6.54	1.40	2.66	0.83	0.90	0.35	3.86	0.42	1.02
Nonacosane (C29)	8.14	27.97	12.42	9.21	8.00	3.76	1.96	31.73	2.12	12.89
**Sum (%)**	***90.22***	***100.00***	***99.17***	***95.26***	***75.54***	***45.38***	***32.27***	***93.29***	***57.49***	***63.49***
**Total amount (ng)**	**266.78**	**123.80**	**206.31**	**119.22**	**118.68**	**834.06**	**1210.49**	**147.82**	**513.69**	**418.86**
										
**Alkene**										
7-Heneicosene	0.00	0.00	0.46	0.00	0.00	0.54	28.43	0.00	0.13	0.35
11-Tricosene	0.00	0.00	0.00	0.00	0.00	0.00	0.00	0.00	0.00	0.16
9-Tricosene	0.00	0.00	0.00	0.00	1.26	1.59	2.41	0.00	1.15	0.63
7-Tricosene	0.60	0.00	0.00	0.00	2.04	2.89	29.03	0.00	0.90	1.02
11-Pentacosene	0.00	0.00	0.00	0.00	0.00	4.43	0.45	0.00	0.00	3.02
9-Pentacosene	0.42	0.00	0.00	0.00	8.50	0.00	0.83	0.00	13.06	0.34
7-Pentacosene	0.80	0.00	0.00	0.00	1.38	13.07	5.31	0.00	2.11	0.42
5-Pentacosene	5.44	0.00	0.00	0.51	1.11	0.99	0.67	0.00	0.44	10.71
11-Heptacosene	0.00	0.00	0.00	0.00	0.00	0.38	0.14	0.00	6.58	8.89
9-Heptacosene	0.78	0.00	0.19	1.62	7.68	6.17	0.00	0.56	10.00	0.86
7-Heptacosene	0.64	0.00	0.00	0.00	1.08	9.02	0.30	1.11	1.15	0.76
5-Heptacosene	0.00	0.00	0.00	0.00	0.00	0.00	0.00	0.00	0.00	0.00
11-Nonacosene	0.15	0.00	0.00	0.00	0.00	0.00	0.00	0.00	3.83	7.95
9-Nonacosene	0.46	0.00	0.19	1.17	1.41	8.18	0.10	3.31	3.18	0.85
7-Nonacosene	0.50	0.00	0.00	1.44	0.00	7.37	0.07	1.74	0.00	0.55
5-Nonacosene	0.00	0.00	0.00	0.00	0.00	0.00	0.00	0.00	0.00	0.00
**Sum (%)**	***9.78***	***0.00***	***0.83***	***4.74***	***24.46***	***54.62***	***67.73***	***6.71***	***42.51***	***36.51***
**Total amount (ng)**	**28.93**	**0.00**	**1.73**	**5.94**	**38.44**	**1003.87**	**2540.77**	**10.64**	**379.86**	**240.84**
										
	*Anacamptis morio*	*Nigritella rhellicani*	*Gymnadenia conopsea*	*Gymnadenia odoratissima*	*Dactylorhiza maculata*	*Himantogl. hircinum*	*Platanthera bifolia*	*Neotinea ustuata*	*Neotinea lactea*	
**Alkane**										
Heneicosane (C21)	0.21	1.98	0.00	10.54	0.00	0.45	0.00	0.55	0.00	
Docosane (C22)	0.15	0.00	0.00	1.61	0.00	0.15	0.00	0.35	0.00	
Tricosane (C23)	10.88	13.21	6.42	39.17	4.03	2.40	1.46	10.34	5.67	
Tetracosane (C24)	3.43	2.33	0.00	1.70	0.00	0.93	1.06	1.45	0.00	
Pentacosane (C25)	38.64	15.57	9.35	14.41	5.35	33.99	18.14	10.52	14.06	
Hexacosane (C26)	3.17	0.00	0.00	1.39	0.00	2.54	2.95	1.59	6.11	
Heptacosane (C27)	28.30	13.44	0.00	0.00	4.78	41.16	31.79	15.13	39.84	
Octacosane (C28)	1.89	0.00	3.38	0.00	2.97	1.29	6.11	1.35	9.75	
Nonacosane (C29)	9.91	4.53	25.44	0.00	33.56	13.23	23.23	7.26	24.56	
**Sum (%)**	***96.58***	***51.06***	***44.59***	***68.82***	***50.69***	***96.14***	***84.74***	***48.54***	***100.00***	
**Total amount (ng)**	***299.09***	***25.20***	***11.80***	***30.87***	***12.89***	***388.24***	***15.96***	***43.35***	***8.87***	
										
**Alkene**										
7-Heneicosene	0.98	0.00	0.00	0.00	0.00	0.42	0.00	0.00	0.00	
11-Tricosene	0.00	0.00	0.00	0.00	0.00	0.00	0.00	42.69	0.00	
9-Tricosene	0.00	0.00	0.00	0.00	0.00	0.09	0.00	0.00	0.00	
7-Tricosene	1.44	0.00	0.00	0.00	0.00	0.22	0.00	0.47	0.00	
11-Pentacosene	0.00	0.00	0.00	0.00	0.00	0.00	0.00	5.42	0.00	
9-Pentacosene	0.00	0.00	0.00	0.00	0.00	0.31	0.00	0.00	0.00	
7-Pentacosene	0.00	0.00	0.00	0.00	0.00	0.65	0.00	0.00	0.00	
5-Pentacosene	1.00	4.15	17.40	12.32	13.85	0.00	0.00	0.00	0.00	
11-Heptacosene	0.00	0.00	0.00	0.00	0.00	0.00	0.00	0.00	0.00	
9-Heptacosene	0.00	5.64	0.00	0.00	0.00	0.52	1.20	0.00	0.00	
7-Heptacosene	0.00	26.08	0.00	0.00	0.00	0.88	0.00	0.00	0.00	
5-Heptacosene	0.00	0.00	33.35	15.69	35.46	0.00	0.00	0.00	0.00	
11-Nonacosene	0.00	0.00	4.65	0.00	0.00	0.00	0.00	0.92	0.00	
9-Nonacosene	0.00	6.54	0.00	0.00	0.00	0.33	9.63	0.00	0.00	
7-Nonacosene	0.00	6.53	0.00	0.00	0.00	0.43	4.43	1.97	0.00	
5-Nonacosene	0.00	0.00	0.00	3.17	0.00	0.00	0.00	0.00	0.00	
**Sum (%)**	***3.42***	***48.94***	***55.41***	***31.18***	***49.31***	***3.86***	***15.26***	***51.46***	***0.00***	
**Total amount (ng)**	**10.60**	**24.15**	**14.67**	**13.99**	**12.54**	**15.58**	**2.87**	**45.96**	**0.00**	

### Evolution of alkenes in Ophrys

The strict consensus tree among the six resulting cladograms (length: 401 steps), with a topology broadly congruent to those reported in previous phylogenetic analyses with broader datasets [40-42], is shown in Fig. 1. As expected, in spite of the fact that the majority of the terminal clades have bootstrap values above 50% (bootstrap values > 50% are shown in Fig. 1), the relationships among the major clades into which the ingroup is split are weakly supported, as observed in previous published analyses [40-42].

The MacClade reconstruction of alkenes production unequivocally indicated alkenes presence as the primitive character state in the group and with only two independent losses in single species (*O. provincialis*, *N. lactea*). Concordantly, Bayesian phylogenetic analysis (data not shown), constrained with the model of nucleotide evolution that fits the data best, (TrN, -lnL = 3378.8591 [43]; gamma distribution shape parameter α = 0.808; base frequencies: freqA = 0.2639, freqC = 0.1830, freqG = 0.2151, freqT = 0.3381; substitution rate: [A-C] = 1.000, [A-G] = 2.425, [A-T] = 1.000, [C-G] = 1.000, [C-T] = 3.919, [G-T] = 1.000), was topologically similar to the tree topology of parsimony analysis and strongly supported the presence of alkenes as the ancestral state for the character.

### Alkenes and pollination

**Figure 2:**
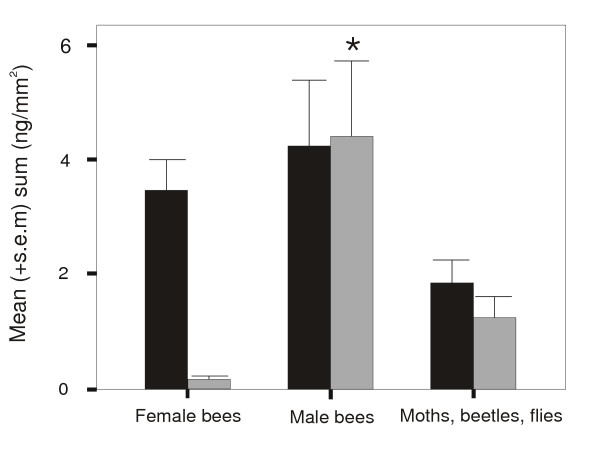
Absolute amounts of alkanes (black bars) and alkenes (grey bars) in orchid groups with contrasting pollinators. Orchids pollinated primarily by male bees (*Ophrys sphegodes*, *Serapias lingua*, *S. cordigera*, *Anacamptis papilionacea) *produce significantly more alkenes (ANOVA, F_2,14 _= 5.89, P = 0.01; see text for posthoc statistics) than the other groups of species. The amounts of alkanes were not significantly different among the pollinator-defined groups.

In our analyses, pollinator category had a significant effect on the absolute amounts of alkenes (Fig. 2; ANOVA, F_2,14 _= 12.05, P = 0.001) and numbers of alkenes (Kruskal-Wallis, Chi^2 ^= 9.0, d.f. = 2, P = 0.01) produced by the flowers. Species pollinated mostly or solely by male bees (*Ophrys sphegodes*, *Serapias lingua*, *S. cordigera*, *Anacamptis papilionacea*; group 2) produced significantly higher absolute amounts of alkenes (LSD Post Hoc, group 1–2: P < 0.001; 2–3 P = 0.002; Fig. 2) and greater number of alkenes with different double bond position (Mann-Whitney U-test: group 1–2: P = 0.01; 2–3: P = 0.006) than the other groups. The "female bees" group was not significantly different from the "moth, beetles, flies" group for amount of alkenes (LSD Post Hoc, P = 0.174; Fig. 2) and number of alkenes (Mann-Whitney U-test, P = 0.84). The amount of alkanes did not differ significantly among the groups (ANOVA, F_2,14 _= 3.69, P = 0.051); however, number of alkanes was different (Kruskal Wallis Chi^2 ^= 6.8 d.f. = 2 P = 0.03), but post-hoc tests did not show any significant differences among the groups.

The relative amounts of alkanes/alkenes were also significantly different between the pollinator categories (ANOVA, F_2,14 _= 21.35, P < 0.001). Comparison among groups revealed all groups to be different from each other (LSD Post Hoc, P < 0.001) except "male bees" and "moth, beetles, flies" (P = 0.17). Species with relatively low absolute amounts but unusually high proportions of alkenes were *Neotinea ustulata*, *Dactylorhiza maculata*, *Gymnadenia conopsea*, *G. odoratissima*, and *G. rhellicani *(Table 2). *Neotinea ustulata *produced unusually high proportions of 11-tricosene and 11-pentacosene. *Dactylorhiza maculata *produced mostly 5-pentacosene and 5-heptacosene. *Gymnadenia conopsea*, *G. odoratissima*, and *G. rhellicani *produced mostly 7- and 5-heptacosene (Table 2).

## Discussion

The evolution of floral odour compounds mediating pollinator attraction is of general interest and can help us to understand evolutionary mechanisms in pollination systems. A relatively well understood pollination system is sexual deception in the European genus *Ophrys*, where mechanisms of pollinator attraction involve the mimicry of alkene patterns of female bees to sexually attract the male pollinators [17]. Until now it was, however, unclear whether the biosynthetic ability to produce these compounds represents an evolutionary novelty of *Ophrys *within subtribe Orchidinae. In our approach, combining phylogeny and chemical ecology, we found that the production of alkenes is widespread among related orchids. Besides that, other species primarily pollinated by male bees produce similar high amounts and diversity of alkenes as *Ophrys*. Thus, alkene production itself is likely a plesiomorphic character in *Ophrys*, having evolved earlier than the pollination syndrome of sexual deception. We suggest that production of alkenes is an example of a preadaptation that evolved from a primary vegetative to reproductive function and enabled the orchids to exploit various behavioural patterns of their pollinators through chemical mimicry.

### Alkenes are preadaptations for sexual deception

The concept of preadaptation in floral traits presumes that existing traits have acquired a new function, which can be in the form of a "transfer exaptation" (new function replaced the old) or "addition exaptation" (new function added to the old) [36]. The identification of floral exaptations is dependent on a combination of phylogenetic analyses and ecological studies investigating functions of floral traits [44]. A well known example is the tropical vine genus *Dalechampia*, where resins are produced primarily for herbivore deterrence [35]; this trait was a preadaptation for the evolution of pollination systems based on resin reward [34,36]. Preadaptations of pollinators have also been assumed to influence the evolution of specialized pollination systems, such as that observed in the yucca – yucca moth mutualism [45]. However, some traits of pollinators in this system are, on the contrary, synapomorphies inferred to be key innovations [46]. For the evolution of sexual deception within Orchidinae, we suggest an evolutionary scenario similar to *Dalechampia*. The ability to produce alkenes, (i.e. the genes encoding the necessary enzymes) is likely an ancestral trait – a preadaptation with vegetative function in the cuticular wax layer. The transition into male-pollinator attraction was achieved by producing increased amounts and different double-bond varieties of alkenes, since female bees produce large amounts of alkenes in specific patterns of double-bond positions [31]. Additionally, morphological changes in the labellum evolved to achieve mimicry of shape, colour and pilosity of female bees [10].

Plants of the genus *Ophrys *generally produce large amounts of different alkenes. Erdmann [47] found alkenes in all 18 analysed species, including *O. speculum*, that uses oxo- and hydroxy acids for pollinator attraction [48]. In this species, even buds and pollinia produce alkenes. We found that large amounts and diversity of alkenes with different double bond positions in *Ophrys *was also shared among other species of Orchidinae, namely the two outcrossing representatives of *Serapias*, and *Anacamptis papilionacea*. Although several evidences, including chromosome number, suggest that *Ophrys*, *Serapias*, and *Anacamptis *are closely related, the exact relationship cannot be inferred by the existing phylogenies that have low bootstrap support for the basal relationship among genera [40,42]. It is thus not clear whether the production of large amounts of alkenes in *Ophrys *is an evolutionary novelty in the genus or inherited from a common ancestor with *Serapias*. Considering the latter possibility, the elaborate morphology of *Ophrys *labella, with often dark color and UV-reflecting "mirror-patterns", that is unique to the genus, should be considered as the key innovation for sexual deception. However, earlier studies suggest that *Serapias *is pollinated by sexual deception as well [49]. Thus, a better understanding of the pollination system in *Serapias*, as well as phylogenies with better resolution of relationships among the major groups are necessary to resolve this issue.

### Alkenes and their role in plant-pollinator interactions

The pattern of strong alkene production in the non-rewarding *Ophrys*, *Serapias*, and *Anacamptis papilionacea *is paralleled by a common use of primarily male bees as pollinators in these orchids. A strong alkene production, as shown by the significantly higher absolute amounts in these species, is the likely prerequisite for these low volatile compounds to act as semiochemicals. Since male bees use alkenes for chemical communication [31], these orchids likely use alkenes to manipulate the behaviour of their pollinators.

In *Serapias*, pollination by male bees sleeping in the flowers has been proposed [50], but earlier studies suggested that males of the bee, *Ceratina cucurbita*, are sexually attracted to the flowers and search for females within them [49]. These flowers may thus mimic either a nest entrance or virgin females sitting in the flowers, to entice males to enter the flowers. The high abundance and diversity of alkenes suggest that alkenes mediate such signalling. *Anacamptis papilionacea *is pollinated by patrolling male bees of the genus *Eucera *[51], and the alkenes produced by the flowers may also render the flowers more attractive for the males. In both species, further investigations are necessary to clarify details of the pollination system and the inferred role of floral odor.

Besides their role as pheromones in bees, alkenes are also important semiochemicals in flies. This may explain the unusually strong production of 11-tricosen and 11-pentacosene in *Neotinea ustulata*, that is pollinated specifically by the tachinid fly, *Tachina *(*Echinomyia*) *mangnicornis *[52]. Many fly species use alkenes as mating signals; one example is *Drosophila virilis*, with 11-pentacosene as its major sex pheromone component [53]. Female flies also use host-pheromones for host location, but little is yet known about the chemical identity of these signals [54]. It is possible that the flower-produced alkenes are also involved in the attraction of the pollinator via false host- or mating signals, and future investigations on the pollination system in this species may also prove interesting. The moderate to high production of alkenes in the rewarding orchid clade (including *Gymnadenia conopsea*, *G. odoratissima*, *G. rhellicani*, *Platanthera bifolia*, together with the nectarless *Dactylorhiza maculata*), may be explained as retention of the primitive state of alkene production.

### Why employ male bees as pollinators?

Male insects invest more time and energy in mating behaviour than females, which spend more time feeding and collecting food for brood care [55]. Therefore, females are generally considered as more efficient pollinators [56]. Whole guilds of plants, however, are specialized for pollination by male bees, e.g. Euglossine pollinated plants that produce fragrance as a reward for the pollinators [57,58], *Oncocyclus *irises being pollinated primarily by night sheltering *Eucera *males [59], and orchids that mimic specific model plants, such as *Cephalanthera rubra *and *Diuris maculata *[60,61]. Since the behaviour of male and female bees differ, the resulting pollination patterns can be assumed to be different, too. Whereas food-seeking female bees are more efficient pollinators, males conduct longer-distance visits and thus contribute relatively more to outcrossing [56]. Indeed, it has been predicted that fragrance-collecting male euglossine bees, as well as males pollinating sexually deceptive orchids, mediate long distance pollen flow [62-65]. As self-compatibility and inbreeding depression are widespread in orchids [66], distance of pollen flow may impact strongly on the quality of seeds produced [67]. Additionally, pollination by exploitation of reproductive behaviour of males is often more specific, particularly in sexually deceptive orchids and orchids pollinated by fragrance-collecting euglossine bees [68,69]. Specificity may decrease pollen loss, a factor that is likely important in orchids, given their highly efficient pollination via pollinia. Collectively, pollination by male bees may be advantageous by mediating specific and long-distance pollen flow, and selection may thus favour floral signals that attract primarily males.

### Mechanisms of attracting males

We suggest that the evolution of male bees as pollinators in the deceptive pollination systems developed by *Ophrys*, *Anacamptis papilionacea *and possibly also *Serapias *was achieved by "sensory exploitation". This concept has been developed in the framework of sexual selection and states that sensory preference in females drives the evolution of male signals [70]. A similar scenario can be proposed for the evolution of pheromone-imitations in response to male pollinators. To attract male pollinators by pseudo-pheromones, the orchids hitchhike on pre-existing, intraspecific communication channels. The existing reception system and behavioural preference of males for alkenes may have enabled the orchids to exploit this pollinator resource relatively easily, by increased production of alkenes, with pre-existing enzyme systems.

## Conclusion

In conclusion, we show that alkenes, the key signal for pollinator attraction in the orchid genus *Ophrys *can be interpreted as a preadaptation for the evolution of sexual deception in the genus. High amounts of alkenes may have evolved earlier in the evolutionary history in other genera for the attraction of male bees as pollinators, through hitchhiking on existing sensory and behavioural preferences of the pollinators. Such parsimonious evolutionary pathways are probably widespread among the diverse chemical communication systems of plants and pollinators. If so, further insights are to be expected in future studies combining phylogenetic and ecological approaches.

## Methods

### Sample collection

For all scent (hexane extracts) collection, fresh, unpollinated flowers were used. Seven species (plants originating from Cilento and Vallo di Diano National Parks, Southern Italy) were kept in the Naples Botanical Gardens in an insect-tight cage; flowers of the other 13 species were collected from wild populations. One labellum of five individuals (if possible) or five labella of one individual (*Serapias cordigera*, *Himantoglossum hircinum*, *Platanthera bifolia*, *Neotinea ustulata*, *Neotinea lactea*) were extracted in 200 μl of hexane (Merck Uvasol) by shaking in a 2 ml vial for 1 min. Afterwards, the labella were removed and the sample stored at -20°C until analysis. Species, sampling locations and their pollination mode are indicated in Table 1 (for the nomenclatural authority of all listed orchid species see [42].

### Phylogeny and ancestral character state reconstruction

Phylogenetic analysis of nuclear ribosomal ITS1 and 2 sequences of the selected taxa was carried out as described in [41] by using *Disa bivalvata *(Diseae, Disinae, Orchidaceae) as outgroup. Ingroup and outgroup species choice was limited to those taxa for which scent data have been collected. Briefly, Genbank available ribosomal sequences were reduced to only ITS1 and ITS2 and alignment was accomplished by using Clustal W with GAPOPEN and GAPEXT parameters set to a value of 4 (for details see [40,41]. The resulting matrix was then subjected to a parsimony analysis by using the software package MEGA 3.2, and bootstrap percentages [71] were calculated with 500 replicates. As a framework for character reconstruction, we used the strict consensus tree obtained in this analysis, and MacClade 4.0 [72] was used to examine alkene production evolution within the clades. MacClade determines character reconstruction by parsimony, or if more than one state can be assigned to branches, MacClade displays an equivocal pattern on those branches [72]. Alkene production was designated as unordered, with no assumptions of transformations between states [72]. To estimate posterior probabilities for individual nodes, a Bayesian phylogeny was inferred using a variant of the Markov chain Monte Carlo (MCMC) algorithm as implemented in the MrBayes software v3.1.2 [73]. Five Markov chains (four heated, one cold) were run for 150,000 generations using random starting trees and fixed model employed in branch length calculations. Trees were sampled every 100 generations. To set prior probabilities for the analysis (stationary frequencies of character states, character substitution rate matrix, proportion of invariable sites and shape parameter of the gamma distribution of the variation), hierarchical likelihood ratio tests (hLRTs) were performed in MODELTEST v3.7 [74] and the best-fitting model given the dataset was chosen.

### Quantitative scent analysis

Before gas chromatographic analysis, 100 ng of octadecane (purity 99.8%, Fluka, Buchs, Switzerland) were added to all samples as an internal standard. One μl of the sample was injected splitless into an Agilent 6890 N gas chromatograph (GC; Agilent Technologies, Palo Alto, USA) equipped with a HP5 column (5% Phenyl-methylpolysiloxane, 30 m × 0.32 mm ∅ × 0.25 μm film thickness, Agilent Technologies). The column was equipped with a 5 m × 0.53 mm diameter deactivated retention gap. The GC was equipped with a flame ionization detector (FID). Hydrogen served as carrier gas (2 ml/min, constant flow mode) and nitrogen was used as make-up gas. The injector temperature was kept at 300°C. The oven was kept at 50°C for one minute and then heated to 300°C at a rate of 10°C per minute and kept at 300°C for 20 minutes. Chromatogram outputs were recorded by the Chemstation program (Agilent Technologies, Palo Alto, USA) for qualitative and quantitative analysis. The internal standard method was applied to calculate absolute amounts of scent compounds. To calculate relative amounts, the absolute amounts of each compound was divided by the sum of all compounds and multiplied by 100.

### Qualitative scent analysis

For identification of compounds, one μl of each sample was injected on column into a Trace GC Ultra with DSQ II mass spectrometer (MS; Thermo Electron Corp., Milan, Italy), equipped with the same column used for quantitative analysis. Helium served as the carrier gas (2 ml/min, constant flow mode). The oven was kept at 45°C (1 min) and then heated to 280°C at a rate of 10°C/min. The transfer line to the MS was heated to 220°C. The ion-source of the MS was heated to 250°C, the MS was run in full scan mode, starting after 5 min; 1.31 scans/s were done at a scan rate of 500.3 amu/s, and the mass range was 50 – 420. Compounds were identified by comparison of mass spectra and retention times with those of synthetic reference compounds. On a HP-5 column, alkenes elute before the corresponding alkanes. Alkenes with the same chain length but different position of the double bond have reproducibly different retention times, so that (*Z*)-11 elutes first, and (*Z*)-5 last. Rather than identifying all compounds present in the extracts, we focussed on straight chain saturated hydrocarbons (n-alkanes) and unsaturated (n-alkenes) hydrocarbons with double bond positions 5, 7, 9, or 11 and of chain lengths 21 – 29. These compounds have been shown previously to be responsible for pollinator attraction in many *Ophrys *species (summarized by [4]). The isomeric configuration of the alkenes was not determined in this study; however, until now, Z-alkenes were primarily found in plant cuticles [21].

### Statistical analysis

For the analysis of the relation between pollination syndrome and hydrocarbon production, the species were classified into three main pollinator categories (Table 1) according to the available information [49,75-77]. The Bee-group was split into female and male, since male bees are important and exclusive pollinators of some orchids. Group (1) "female bees", comprised only deceptive species, with generalized food deception being predominant. Group (2) "male bees", comprised sexual deception (*Ophrys*), sleeping hole (the outcrossing *Serapias *spp.), and pollination by patrolling *Eucera *males (*A. papilionacea*). Group (3) "moths, beetles, flies", with both rewarding and deceptive species. For most species, these categories should be seen as quantitative estimations, since few Mediterranean orchids are highly specialized [77]. Two species were excluded from the analysis: *Serapias parviflora*, which is an autogamous species [78], and *Neotinea lactea*, where the pollinators remain unknown. For the comparison of absolute amounts of alkane/alkene, all amounts were divided by the number of labella sampled, to normalize the amounts to a single labellum. The size of the labellum was estimated, by using published data on average length and width of labella [79], calculating the product and dividing it by two (Table 1). The absolute amounts of alkane and alkene were normalized to the size of the flowers by dividing it by this value for labellum size. The resulting values were ng/mm^2^. An ANOVA with LSD post-hoc tests was used to compare absolute amounts of alkanes/alkenes among pollination systems. A Kruskal-Wallis test with Mann-Whitney U-test for post-hoc comparisons with the level of significance set to 1% (Bonferroni correction) was used to compare the number of alkanes/alkenes produced.

## Authors' contributions

FPS did the sampling, floral scent analysis and wrote the paper. SC did the phylogenetic analysis and the reconstruction of ancestral state. Both authors contributed to the discussion of the results.
